# Measuring behaviour in hens using an ethogram to assess analgesia during further refinement of a high welfare, on-hen, poultry red mite feeding device

**DOI:** 10.12688/f1000research.133390.1

**Published:** 2023-06-20

**Authors:** F. G. Nunn, D. A. Ewing, K. Bartley, Javier Palarea-Albaladejo, W. Chen, D. R. G. Price, A. J. Nisbet

**Affiliations:** 1 Vaccines, Moredun Research Institute, Penicuik, Scotland, EH26 0PZ, UK; 2 Biomathematics and Statistics Scotland, Edinburgh, EH9 3FD, UK

**Keywords:** ethogram, poultry, EMLA, mite, refinement, behaviours, welfare

## Abstract

**Background**: To refine an on-hen mite feeding device, an ethogram was employed to measure the reactions of hens during a routine experimental procedure (feather plucking) and to assess effects of analgesic cream on those reactions.

**Methods**: Three experimental groups were used; one treated with EMLA 5% before plucking (“EMLA group”); one with aqueous cream (“placebo group”) and a “no treatment” group. Behaviours were measured and compared on three days: ‘dummy handling day’ i.e. no plucking; ‘plucking day’, plucking the left thigh; and ‘treatment day’ i.e with right thighs plucked post-treatment. Poultry red mite feeding assays were performed to examine effect of creams on mite feeding rates, mortality and fecundity. All data were analysed using generalised linear (mixed) modelling approaches.

**Results**: Use of the ethogram demonstrated no significant difference in hen behaviours in the EMLA group between dummy handling day and treatment day (p = 0.949) alongside a significant reduction in measured behaviours between plucking day and treatment day in the same group (p = 0.028). There was a statistically significant increase in measured behaviours from the dummy handling day to the plucking day in both placebo (p = 0.011) and no treatment group (p < 0.001). Effect sizes and directions were similar between dummy handling and treatment days in the ‘placebo’ and ‘no treatment’ groups, though not statistically significant (placebo, p = 0.064; no treatment p = 0.069). Mite feeding in the EMLA group was significantly lower than in the no treatment group in feeding assay 1 (p = 0.029) only. Mite mortality and fertility were unaffected.

**Conclusions**: The ethogram successfully measured changes in observed behaviours between the dummy handling session and procedures. No adverse effects of EMLA cream on hens were demonstrated at 3mg/kg in hens. Use of analgesia for this routine procedure improves hens’ experiences during experimental trials.

## Introduction

**Figure 1.**
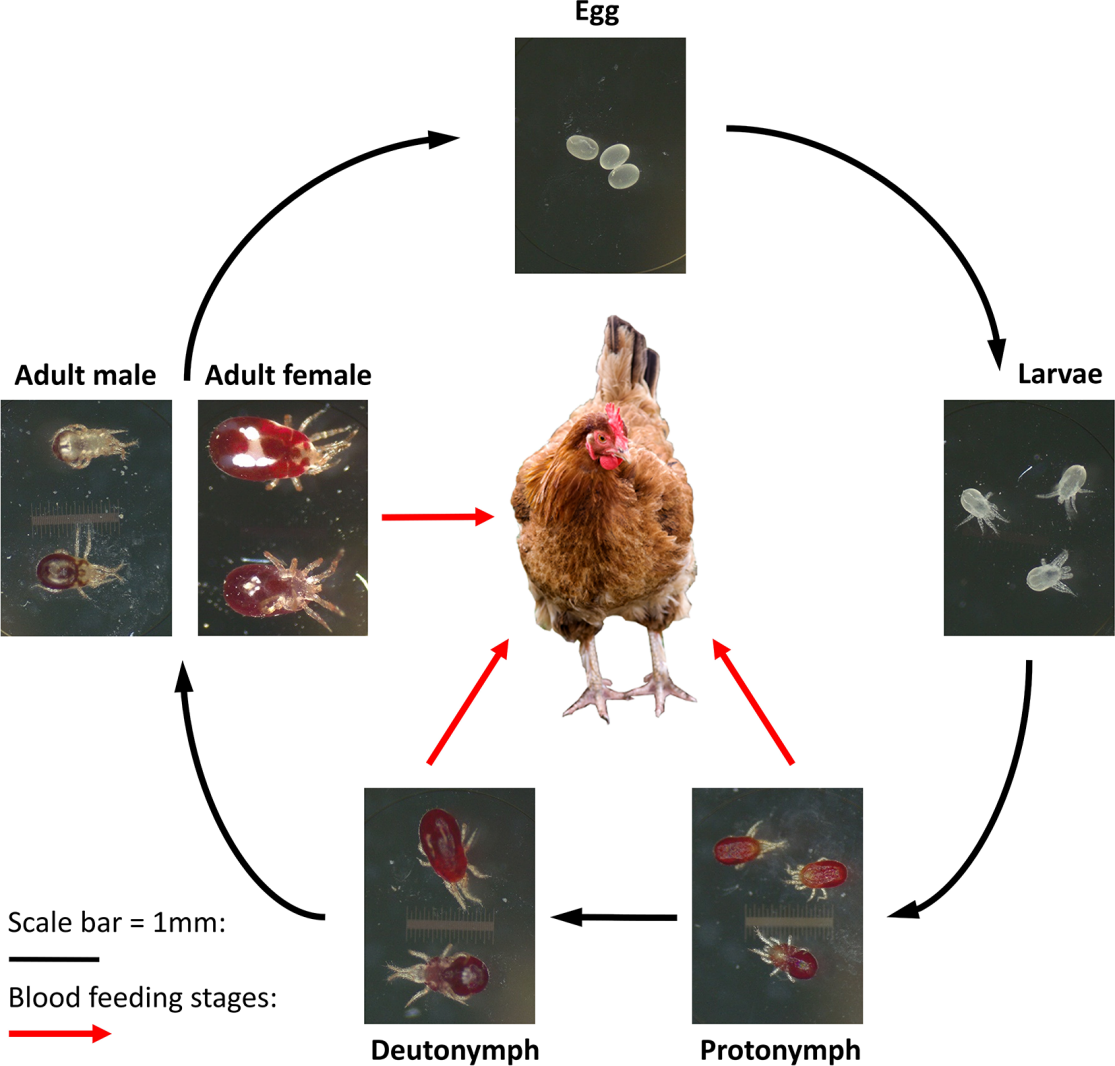
Life cycle of *Dermanyssus gallinae* showing the five life stages of the mite. Blood feeding stages are indicated with the red arrows, with both protonymphs and deutonymphs requiring a feed in order to moult into the next life stage.

Poultry red mite (PRM) is a major pest of laying hens with a worldwide distribution ( Sigognault Flochlay *et al.,* 2017) and a major welfare concern. The mite has a simple life cycle ( Figure 1) and infestations cause stress behaviours, a decrease in egg production, egg quality and an increase in mortality ( Sigognault Flochlay *et al.,* 2017). Research approaches include vaccine development and systemic acaricides which require testing on-hen, using various hen infestation models ( Bartley *et al.,* 2017; Mul *et al.,* 2015). In 2019, we reported the development of a high-welfare, on-hen mite feeding device ( Nunn *et al.*, 2019) that allowed all hematophagous PRM life stages to feed on-hen. This is an important refinement on other methods of testing and allows a reduction in the number of birds needed to test a novel control product ( Nunn *et al.,* 2019). The device has now been used to assess several PRM vaccine candidates ( Price *et al.,* 2019; Lima-Barbero *et al.*, 2019a, b).

Use of this device requires plucking of the hens’ thighs because close attachment of the feeding mesh to the skin facilitates mite feeding. Plucking causes transient pain/distress. The use of analgesic cream may relieve this pain/distress but there is a paucity of published information on the effectiveness of such creams on birds ( Machin, 2005; Hocking *et al.*, 1997). Equally, there is little published information on the assessment of pain in birds. Flecknell (1994) argues that instead of focusing on detection of pain, we should strive to alleviate both pain and distress due to the difficulty in differentiating between the two in animals.

The purpose of the current study was to ascertain if an ethogram used to measure behaviours could gauge discomfort and/or distress in hens during a routine procedure and to assess whether the use of EMLA cream afforded any measurable relief from pain/distress during the same procedure. In addition, respiration rate was assessed as a measurable effect of discomfort/distress. PRM feeding assays were performed on the three days following treatment to assess any increased mortality or reduced feeding rates of mites fed on hens treated with EMLA and placebo creams.

### Ethical considerations

All efforts were made to ameliorate any suffering of hens used in this study. Staff were experienced with the feeding assays and procedures and the veterinary grade bandages and tape have been previously used with no harm caused. Hens were kept in floor pens in groups with pens divided by wire so that each group could see all the other groups and all treatments were carried out in a different room where birds could not see the other birds. Birds always had access to pellets and water, with nest boxes and perches available. The pens were based within animal accommodation with clear panels in the ceiling allowing for natural light. Birds were monitored several times daily and constantly throughout ‘treatment day’ for any adverse signs of EMLA cream. Potential adverse signs included ataxia, distress or recumbency which would result in veterinary intervention, although were understood from the available literature to be transient. No humane endpoints were anticipated from the mite feeding assays outside any sudden ill-health.

All experimental procedures described here were reviewed and approved by the Moredun Research Institute Animal Welfare Ethical Review Body (7/04/2021) and were conducted under the legislation of UK Home Office Project License (reference P46F495BD) in accordance with the Animals (Scientific Procedures) Act of 1986. The manuscript was written in adherence with the ARRIVE 2.0 guidelines ( Percie du Sert *et al.*, 2020; Nunn, Francesca, 2023).

## Methods

### Hens

Bovans Hyline pullets (n=15, 17 weeks old) were purchased from a commercial hatchery and haphazardly assigned to floor pens (3×4 hens and 1×3 hens) by animal care staff. Pens contained perches, nest boxes, substrate in the form of wood shavings and straw and *ad libitum* pellets and water. Hens were permitted to acclimatise for 7 days before the start of procedures.

### Parasite material

Poultry red mites were collected and pre-conditioned as previously described ( Nunn *et al.,* 2019, 2020). In short, mites were collected from farms by gently scraping mite aggregations with a spatula into a weigh boat and then transferring these into a 75 cm ^3^ tissue flask. Mites were then stored in 75 cm ^3^ tissue flasks (Corning, New York, USA) with vented caps and stored at room temperature (RT) for 7 days before being kept at 5°C for three weeks. Adult female mites were isolated on ice as described in Nunn *et al.* (2020) for use in this trial. Briefly, flasks containing mites were placed at RT for 15 minutes. Mites that crawled up into the cap during this time were tapped out onto a glass petri dish and the dish was then placed on a weigh boat containing wet ice. Females were identified using size under light microscopy, counted and placed into pouches using fine detail paint brushes.

### Experimental design

**Trial**

A timetable of the experiment can be seen in Table 1. Three experimental groups consisted of; the “EMLA group” which had EMLA (5%) cream applied on treatment day, the “placebo group” which had aqueous cream (Lloyds Pharmacy Aqueous cream B.P.) applied on treatment day (to facilitate blinding of the groups to the plucker and ethogram scorer) and the “no treatment group”, which had no creams applied on treatment day. The no treatment group existed to facilitate a real-time mite feeding control group to enable comparisons of feeding, mortality and fecundity of mites not exposed to EMLA or aqueous cream. No *a priori* exclusion criteria were set for this study.

**Table 1.** Timeline of trial. Day 1 began seven days after hens were acquired to allow for acclimatisation. Hen handling was carried out by experienced researchers throughout. All hens (n=15) were habituated to handling and a one-minute restraint on day 1 and day 2 in the treatment room. On day 3 all hens (n=15) were handled, with baseline behavioural ethogram recordings, respiratory rates and hen weight recorded. On day 6, all hens (n=15) were taken into the treatment room, restrained and a respiratory rate taken prior to having a 2×2 cm area of their left thigh plucked and behaviour scores were taken. A further respiratory rate was taken straight after plucking was finished. On day 7, hens either had EMLA cream (n=5) or aqueous cream (n=5) applied to a 2×3 cm area on their right thigh with bandages put on top or bandages only (n=5), before being returned to their pens. Hens were then retrieved, bandages removed, respiration rate taken and an area of 2×2 cm plucked with behaviour scores recorded. Respiratory rate was taken immediately after plucking was finished. On days 8, 9 and 10, each hen had a pouch containing 50 female mites attached to their right thigh with medical tape for three hours before its removal.

Day 1	Day 2	Day 3	Day 6	Day 7	Day 8	Day 9	Day 10
Habituation to handling	Habituation to handling	Dummy handling-ethogram scoring, respiration rates	Plucking left thighs - ethogram scoring, respiration rates, weighing	Treatment application on right thighs, plucking right thighs, ethogram scoring, respiration rates	Mite feeding assay 1 - feeding rates, mortality, fecundity over 6 days	Mite feeding assay 2 - feeding rates, mortality, fecundity over 6 days	Mite feeding assay 3 - feeding rates, mortality, fecundity over 6 days

Ethogram behaviours and respiration rates were measured on three days as follows; dummy handling day (i.e. with no plucking), plucking day (all left thighs plucked with no treatment) and treatment day (right thighs plucked with treatments in the EMLA and placebo group). Poultry red mite feeding assays took place on the three days after the treatment day.

The trial was designed so that both ‘plucker’/ethogram scorer and bird handler were blinded to the EMLA and placebo groups throughout the trial including recording of mite feeding rates and mite mortality monitoring. The ethogram scorer also applied the treatments, wearing gloves (Protech), changing pairs between each bird.

### Ethogram design

The behavioural ethogram (see the *Underlying data*, Nunn, 2023) was designed by handlers experienced in the procedure and included all observed reactions to plucking from previous trials. Presence or absence of each behaviour in response to plucking or handling was recorded by scoring 1 (reaction observed) or 0 (no reaction observed), respectively. The behaviours included in the ethogram were; blinking corresponding with plucking, looking at leg, trying to move leg, bodily resistance (wing), vocalisation (once), vocalisation (repeated), vocalisation (loud), and trying to stand/flee. Vocalisation was only scored if it occurred in response to a feather being manipulated (as in ‘dummy handling’) or to a feather being plucked, which is distinct from general background vocalisation.

### Behaviour and respiration rate recordings

After the acclimatisation period, hens were habituated to handling by removal from pens, being carried to the treatment room and then restrained in the same way as they would during procedures, for one minute for two consecutive days ( Table 1, days 1 and 2). All raw behavioural data, respiration data and information on bird weights, doses, treatment groups and pen distribution can be found in the *Underlying data* ( Nunn, 2023).

On day 3, baseline recordings were made during a ‘dummy handling’ session. Individually, each hen was taken from their pen to the treatment room and given one minute to recognise their surroundings. Hens were then restrained on their side and respirations recorded for 30s. Respirations were recorded by gently laying a finger on the keel bone and the number of times the finger was raised was counted within the 30s. To take behaviour and respiratory measurements that were related to hen handling but not plucking, a 60s period of gentle feather manipulation was then used to score behaviours according to the ethogram. This was immediately followed by a second respiratory rate taken over 30s. Hens were then assigned a pre-numbered leg ring and the number recorded next to their behaviour and respiration rate recordings and returned to their pen.

On day 6 (“plucking day”), hens were again selected from each pen, taken to the treatment room and respiratory rate measured. Leg ring numbers were obscured from plucker and scorer by turning the number away from the handlers. A section of thigh approximately 3 cm×3 cm on the left leg of each hen was then plucked and the behaviour for each bird was scored. A second respiration rate was then recorded and birds were then weighed and returned to their pen.

On day 7 (“treatment day”), hens were assigned a treatment group with treatment groups being intermingled across the four pens and with birds chosen to be as close to the average weight of the group as possible. Doses of EMLA cream for each bird were calculated by weight and placebo cream (aqueous cream) doses were the equivalent of the EMLA cream doses by weight. Hens were selected from each pen, taken to the treatment room and respiratory rate measured. Treatments were applied using a child’s interdental brush (Microbrush ^®^ Plus Micro Applicator regular size, PR400GR, Microbrush Corporation, USA) to gently apply cream around the base of the feathers on the hens’ right thighs until the cream was uniformly distributed in the 3×3 cm area where the feeding device would be subsequently attached (days 8–10). A cotton gauze swab (Covetrus 10 cm×10 cm) was placed on top and was then bandaged with a cohesive bandage (Steroban Cohesive, 2.5 cm×4.5 m). The no treatment group had the cotton swab and bandage applied only. Hens were then placed back in their respective pens. So that all treatments could be left on the birds for a set period, an electronic timer was set at time = 0 when the first bird had a treatment and the pens were completed to facilitate plucking at around 45mins after treatment application. There was no evidence to suggest a difference between application periods across the treatment groups based on Tukey’s multiple comparisons of means, with the following estimated differences (in minutes) between groups; “no treatment – EMLA” 2.0 (-3.0, 7.0), “placebo – EMLA” 0.6 (-4.4; 5.6) and “placebo – No Treatment” -1.4 (-6.4; 3.6). Birds were continually monitored during this application period for any adverse effects such as loss of balance or distress (none were observed). Hens were then brought into the treatment room in order of treatment application, respiratory rate was measured and bandages removed, right thighs plucked and behaviour recorded before the respiratory rate was then measured again. ^1^

### Poultry red mite feeding assays

Feeding assays were carried out as previously reported ( Nunn *et al.*, 2019, 2020). Briefly, each hen was haphazardly allocated a feeding device on each of the three days after treatment day. Pouches containing 50 adult female PRM were applied to the hens’ right thighs and attached with medical tape for three hours. At the end of the feeding period, hens were haphazardly selected for removal of the feeding devices. Pouches were then removed with “fed” and “unfed” mites recovered and counted to assess feeding rates. Fed mites were kept for observing mortality and fecundity by counting eggs laid per fed female, over a 6-day period.

### Power calculations

Power calculations were based on simulation from a binomial generalised linear mixed model (GLMM) for mortality and feeding rates over time as described below and included in the *Underlying data* ( Nunn, 2023).

**Mortality rates.** We assumed a mortality rate of 2% as baseline and increases of 5% and 7% that would be considered biologically meaningful differences in mortality rates after the application of topical analgesia, three time points, 50 mites per feeding pouch, and normally distributed random effects for time (SD=0.35) and bird (SD=0.14). Model parameters were based on previous data from PRMs in all life stages fed on hens of the same age ( Nunn *et al.,* 2020).

**Feeding rates.** We assumed a feeding rate of 65% as baseline and reductions to 60% and 55% that would be considered biological meaningful reductions after the application of topical analgesia, three time points, 50 mites per feeding pouch, and normally distributed random effects for time (SD=0.23) and bird (SD=0.11). Model parameters were based on previous data (see the *Underlying data* ( Nunn 2023); Data: Obj 2 18 weeks data for Power Calcs) from PRMs in all life stages.

The results indicated that five birds per experimental group was the minimum required to reach the standard 80% statistical power threshold at a 5% significance level for both mortality rates and feeding rates under those conditions.

No power calculations were performed for the behaviour/ethogram analyses or for respiration rates. Due to the nature of mixed model analysis reference to experimental units are not made, as mixed models have multiple random components with affects assessed against appropriate levels in the random hierarchy.

### Statistical analysis of behaviour scores, respiration rates and mite fertility, mortality and deeding

The behaviour scores were analysed using Firth’s bias-reduced logistic regression ( Firth, 1993), to allow for combinations of treatment and timepoint with entirely zero responses, with the binary response variable denoting whether a behaviour was observed or not. A 3-way interaction between the treatment group, behaviour type (as described above) and the day was considered, alongside all 2-way interactions and main effects. There was little evidence of variability between birds or pens, so random effects were not included in the model.

Respiration rates were analysed using generalised linear mixed models (GLMMs) with a Poisson response and logarithmic link function fitted by maximum likelihood to the respiration counts. A 3-way interaction between the treatment group, whether the measurement was pre- or post-treatment, and the day was considered, alongside all 2-way interactions and main effects. Random effects were included for each bird but there was no evidence to support a more complex structure (e.g. random effects for pen or day nested within bird).

Analysis of mite feeding rates was conducted using GLMMs, with a binomial response and a logit link function, fitted by maximum likelihood to the number fed out of total mites per feeding pouch (i.e. per bird by assay) including treatment group and assay number and the interaction between these two terms as fixed effects and bird as a random effect (there was no evidence of a pen effect). Equivalent models were considered for the mite mortality rates, however no variance was attributed to bird or pen so generalised linear models (GLMs) with the same fixed effects structure were fitted to the number dead after 6 days out of total fed mites. Fertility rates of mites (i.e. total offspring per fed mite), were analysed using a zero-inflated Poisson GLMM fitted to the number of offspring per pouch with a logarithmic link function, including an offset (on logarithmic scale) to account for total number of fed mites recorded per pouch, with the interaction between treatment group and assay number as a fixed effect and bird as a random effect.

Assessment of the statistical significance of fixed effects was conducted using likelihood ratio tests. The use of mixed models ensures that fixed effects were assessed against appropriate variances in the random hierarchy. Post hoc pairwise tests of differences in means were conducted based on the predicted marginal means from the model estimates. The resultant p-values were adjusted to account for multiple testing using the multivariate t-distribution. Results show contrast effect sizes (es) and standard errors (SE) on the transformed scales (e.g. log, logit). Means and 95% confidence intervals (LCL, UCL lower and upper limits of the 95% confidence interval, respectively) estimated from the above models back transformed onto the original scale (e.g. proportions) are also shown to aid interpretation. Significance tests were assessed at the standard 5% significance level. All analyses were conducted using R (4.1.3., R Core Team, 2022) with the lme4 package for GLMMs ( Bates *et al.*, 2015), the glmmTMB package for zero-inflated models ( Brooks *et al.,* 2017), the brglm package for Firth’s bias-reduced logistic regression ( Kosmidis, 2021), the car package for likelihood ratio tests ( Fox and Weisberg, 2019) and the emmeans package for pairwise comparisons and multiple testing adjustments ( Lenth, 2022).

## Results

### Behaviour comparisons for experimental groups

Summaries of the dose rates, application duration and weights of birds are shown in Table 2. Raw data for behaviour and respiration scores, experimental groupings, dose weights etc are in the *Underlying data* ( Nunn, 2023).

**Table 2.** Showing mean weight (kg), dose (mg) and the time of application (min) across the three treatment groups (n=5). The standard deviations are given in brackets for each group.

Treatment	Weight (kg)	Dose (mg) at 3 mg/kg	Time left (min)
EMLA	1.49 (0.13)	89.3 (7.9)	44.8 (1.6)
Placebo	1.38 (0.08)	83.0 (4.8)	45.4 (2.1)
No treatment	1.46 (0.06)	87.4 (3.7)	46.8 (4.4)

**Figure 2.**
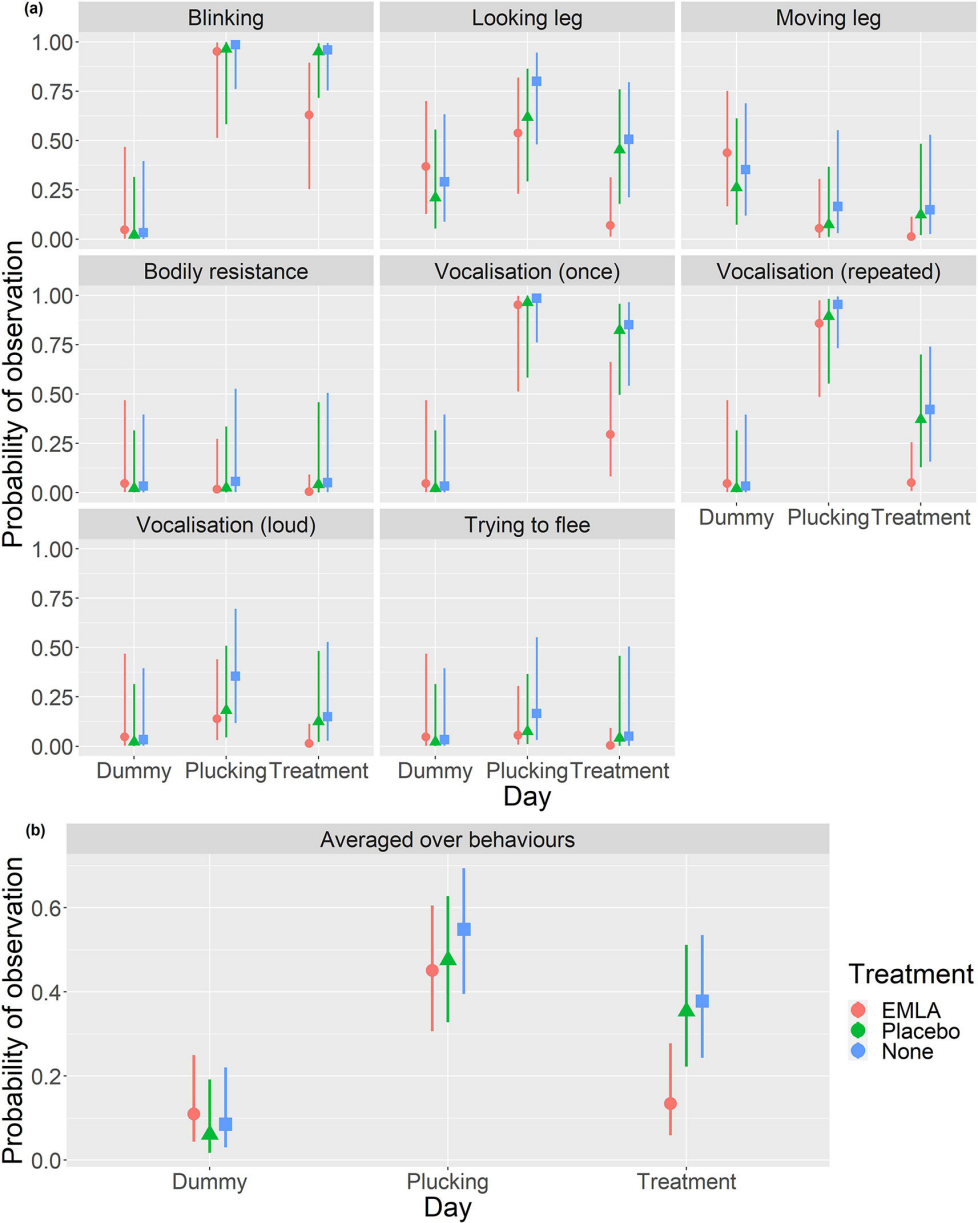
Behavioural results from the EMLA group ( *n* = 5), placebo group, ( *n* = 5) and no treatment group ( *n* = 5) during dummy handling day, plucking day and treatment day. Plots in the top panel (a) show the estimated probability of each behaviour being displayed with 95% CI for each combination of day and treatment group. The bottom panel (b) shows the estimated probability with 95% CI of observing a pain related behaviour averaged across behaviour types for each treatment group on each day (generating estimates in Table 3a and comparisons in Tables 3b and 3c).

When analysing the ethogram scores the best fitting model included 2-way interactions between the behaviour type and the day (p<0.001) and between the treatment and day (p=0.053) alongside main effects for each of the terms. Averaged over behaviours ( Table 3a and Figure 2b), no statistically significant differences between any of the three treatment groups were observed on either the dummy handling or plucking days ( Table 3b). There was evidence of a statistically significant decrease in the probability of observing a pain-related behaviour in the EMLA group compared to either the placebo (es=-2.41, p=0.040) or no treatment (es=-2.62, p=0.020) groups on the treatment day ( Table 3b). Figure 2a shows that these differences appear to be driven primarily by a subset of the measured behaviours; namely blinking, looking at the leg and vocalisations.

**Table 3.** (a) Shows the estimated probability of observing a pain-related behaviour (averaged across all of the behaviour types) for each combination of treatment group (n=5) and day (used to generate Figure 2b) from the Firth’s logistic regression. (b) and (c) Show the estimated differences between treatment groups within days (b) and between days within treatment groups (c) in the mean probabilities (on the logit scale) of observing a pain-related behaviour across the treatment groups and timepoints with p-values adjusted for multiple comparisons within each table. “SE”, “LCL” and “UCL” are the standard error, and the lower and upper limits of the 95% confidence interval, respectively.

(a)				
Group	Day	Probability	LCL	UCL
EMLA	Dummy	0.11	0.04	0.25
Placebo	Dummy	0.06	0.02	0.19
None	Dummy	0.09	0.03	0.22
EMLA	Plucking	0.45	0.31	0.61
Placebo	Plucking	0.48	0.33	0.63
None	Plucking	0.55	0.39	0.69
EMLA	Treatment	0.13	0.06	0.28
Placebo	Treatment	0.35	0.22	0.51
None	Treatment	0.38	0.24	0.54

(b)					
Contrast	Day	Estimate	SE	z.ratio	p.value
EMLA – Placebo	Dummy	0.78	0.83	0.95	0.940
EMLA – None	Dummy	0.35	0.77	0.46	0.999
Placebo - None	Dummy	-0.43	0.85	-0.51	0.998
EMLA – Placebo	Plucking	-0.33	0.76	-0.43	0.999
EMLA – None	Plucking	-1.23	0.77	-1.60	0.568
Placebo - None	Plucking	-0.91	0.75	-1.21	0.832
EMLA – Placebo	Treatment	-2.41	0.86	-2.81	0.040
EMLA – None	Treatment	-2.62	0.86	-3.03	0.020
Placebo - None	Treatment	-0.21	0.64	-0.33	1.000

(c)					
Contrast	Treatment	Estimate	SE	z ratio	p value
Dummy – Plucking	EMLA	-1.90	0.83	-2.30	0.160
Dummy – Treatment	EMLA	0.85	0.94	0.90	0.949
Plucking – Treatment	EMLA	2.75	0.94	2.90	0.028
Dummy – Plucking	Placebo	-3.01	0.93	-3.20	0.011
Dummy – Treatment	Placebo	-2.34	0.88	-2.60	0.064
Plucking – Treatment	Placebo	0.67	0.77	0.90	0.958
Dummy – Plucking	None	-3.48	0.86	-4.00	<0.001
Dummy – Treatment	None	-2.12	0.81	-2.60	0.069
Plucking – Treatment	None	1.36	0.75	1.80	0.414

There was a statistically significantly lower probability of exhibiting a pain related behaviour in the EMLA group on treatment day than on plucking day (es=2.75, p=0.03) and no statistically significant evidence of a difference between treatment day and the dummy handling day (es=0.85, p=0.95) ( Table 3c). In the other two treatment groups there was a statistically significant increase in the probability of recording a behaviour on plucking day compared to dummy handling day (placebo; es=3.01, p=0.011, no treatment; es=3.48, p<0.001) but no statistically significant difference between the response probabilities on the other days. Nonetheless, it should be noted that there were fairly similar differences between the dummy handing and treatment days as between the dummy handling and plucking days but these were marginally statistically insignificant at the 5% level ( Table 3c).

### Respiration change for experimental groups

The respiration rates and percentage changes in respirations following handling summarised by treatment group and day are shown in Table 4. Following model selection there was no evidence of a statistically significant effect of the treatment group on the respiration rate before or after treatment, with the significant term being the effect of day (p=0.018), which stemmed from a statistically significantly higher respiration rate on the plucking day than on the dummy day (es (log respiration rate)=0.18, p=0.013).

**Table 4.** Respiration rates (mean counts in 30 secs with standard deviations given in brackets) pre and post procedure (handling) by group (n=5) including percentage respiration change during dummy handling, plucking day and treatment day.

Treatment	Day	Pre-handling	Post handling	% change after handling
EMLA	Dummy	15.2 (3.8)	14.6 (4.6)	-3.6 (15.8)
EMLA	Plucking	16.8 (4.7)	18.8 (6.5)	10.1 (8.3)
EMLA	Treatment	15.4 (5.1)	15.6 (5.0)	1.7 (3.7)
Placebo	Dummy	16.6 (1.3)	15.2 (2.2)	-8.8 (5.8)
Placebo	Plucking	16.0 (2.2)	18.6 (2.1)	16.7 (6.8)
Placebo	Treatment	15.2 (2.6)	17.6 (2.7)	16.6 (11.6)
No treatment	Dummy	15.8 (1.6)	15.0 (2.2)	-5.1 (10.4)
No treatment	Plucking	19.0 (2.2)	21.2 (2.7)	11.6 (6.0)
No treatment	Treatment	18.4 (2.9)	20.2 (2.7)	10.2 (5.7)

### Comparison of poultry red mite feeding rates by treatment group and assay

**Figure 3.**
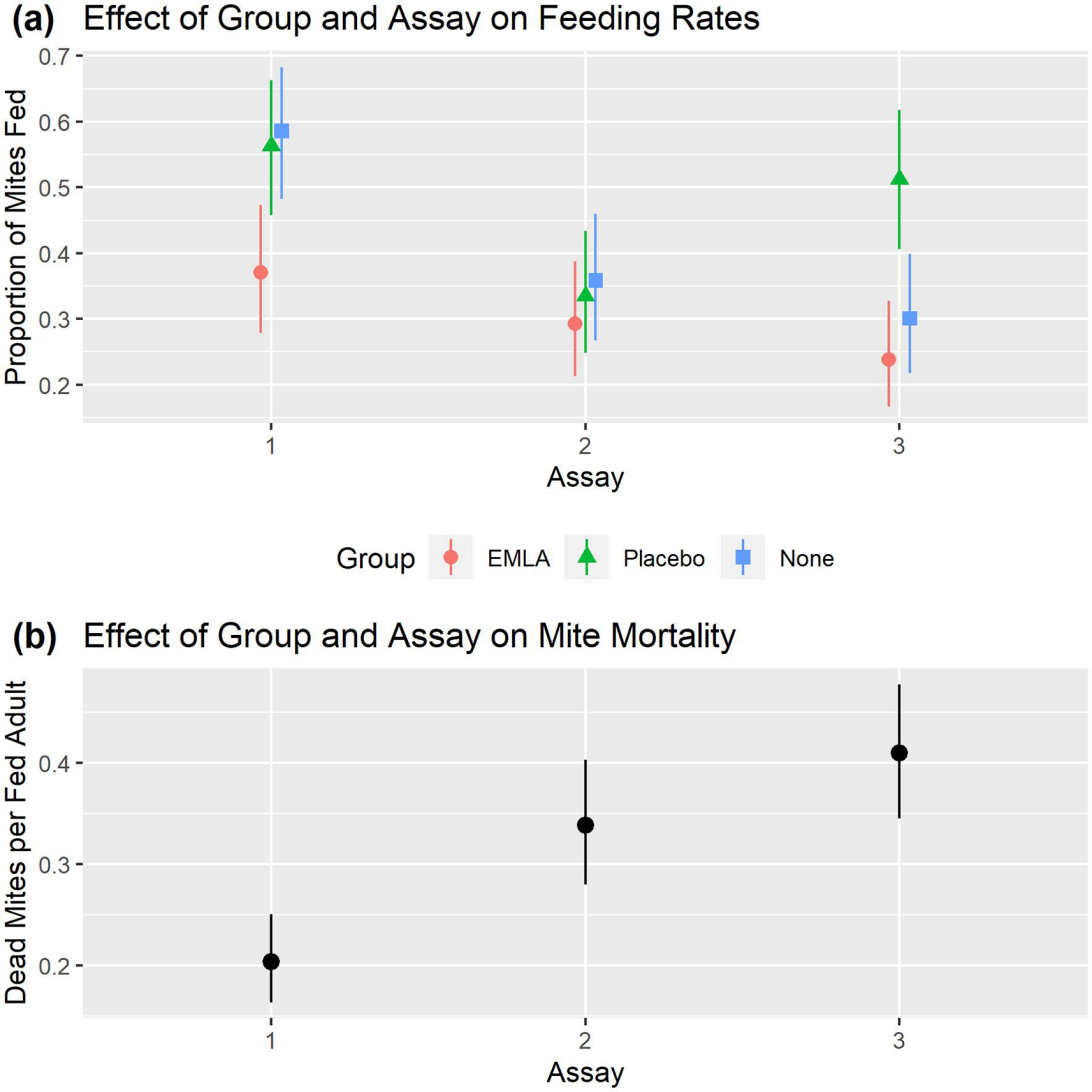
Mite feeding and mortality rates for the EMLA group (n=5), placebo group (n=5) and no treatment group (n=5) for the three assays. Plots in the top panel show the estimated proportion of mites fed with 95% CI for each combination of treatment group and feeding assay (generating estimates in Table 5a and comparisons in Tables 5b). The bottom panel (b) shows estimated mite mortality rates with 95% CI of fed mites for each combination of treatment group and feeding assay (generating estimates in Table 6a and comparisons in Table 6b).

Raw data is in the *Underlying data* ( Nunn, 2023). For feeding rates, there were statistically significant main effects of treatment group (p=0.017) and assay number (p<0.001) and a statistically significant interaction between these effects (p<0.001). The estimated mean mite feeding rate ( Figure 3a and Table 5a) was always lowest in the EMLA group, irrespective of assay and was statistically significantly lower ( Table 5b) in the EMLA group than in the group with no treatment applied in the first assay (es=0.88, p=0.029). The estimate of the difference between the EMLA group and the placebo group was similar to the difference between the EMLA group and no treatment group in assay 1, though not statistically significant at the 5% level (es=0.78, p=0.070). There was no evidence of a statistically significant difference between the groups in assay 2. In assay 3 the feeding rate was statistically significantly higher in the placebo group than in either the EMLA group (es=1.21, p=0.001) or the group which received no treatment (es=0.90, p=0.031), however there was no difference between the EMLA group and the group which received no treatment (es=0.32, p=0.879).

**Table 5.** (a) Shows estimated proportion of poultry red mite (PRM) that have fed (i.e. number of fed adult mites as a proportion of the total number of mites recorded) for each combination of treatment group and assay number (used to generate Figure 3a) from the generalised linear mixed model with binomial response. (b) Shows the estimated differences between treatment groups within assay numbers in the mean probabilities (on the logit scale) with p-values adjusted for multiple comparisons. “SE”, “LCL” and “UCL” are the standard error, and the lower and upper limits of the 95% confidence interval, respectively.

(a)					
Group	Assay	Proportion	SE	LCL	UCL
EMLA	1	0.37	0.050	0.28	0.47
Placebo	1	0.56	0.053	0.46	0.66
None	1	0.59	0.052	0.48	0.68
EMLA	2	0.29	0.045	0.21	0.39
Placebo	2	0.33	0.048	0.25	0.43
None	2	0.36	0.050	0.27	0.46
EMLA	3	0.24	0.041	0.17	0.33
Placebo	3	0.51	0.055	0.41	0.62
None	3	0.30	0.047	0.22	0.40

(b)					
Contrast	Assay	Estimate	SE	z ratio	p value
EMLA - Placebo	1	-0.78	0.30	-2.58	0.070
EMLA - None	1	-0.88	0.30	-2.89	0.029
Placebo - None	1	-0.09	0.30	-0.30	0.999
EMLA - Placebo	2	-0.20	0.30	-0.64	0.984
EMLA - None	2	-0.30	0.31	-0.97	0.894
Placebo - None	2	-0.10	0.30	-0.34	0.999
EMLA - Placebo	3	-1.21	0.32	-3.85	0.001
EMLA - None	3	-0.32	0.32	-1.01	0.879
Placebo - None	3	0.90	0.31	2.87	0.031

### Comparison of mite mortality by treatment group and assay number

The assay number was found to have a statistically significant effect on the mite mortality at the 5% level (p<0.001), however there was no statistically significant effect of either the main effect of treatment group (p=0.100) or the interaction between assay and treatment group (p=0.230). The mite mortality rate ( Figure 3b and Table 6a) was statistically significantly lower ( Table 6b) in the first assay than in either the second (p=0.001) or the third (p<0.001) assays, however there was no statistically significant difference between the second and third assays (p=0.278).

**Table 6.** (a) Shows estimated proportion of poultry red mite (PRM) that died (i.e. recorded number of dead adult mites as a proportion of the total number of fed mites recorded) for each assay number (used to generate Figure 3b) from the generalised linear mixed model with binomial response. (b) Shows the estimated differences between assay numbers in the mean probabilities (on the logit scale) with p-values adjusted for multiple comparisons. “SE”, “LCL” and “UCL” are the standard error, and the lower and upper limits of the 95% confidence interval, respectively.

(a)				
Assay	Proportion	SE	LCL	UCL
1	0.20	0.022	0.16	0.25
2	0.34	0.032	0.28	0.40
3	0.41	0.034	0.34	0.48

(b)				
Contrast	Estimate	SE	z ratio	p value
1-2	-0.69	0.20	-3.5	0.001
1-3	-1.00	0.19	-5.1	<0.001
2-3	-0.30	0.20	-1.5	0.278

### Mite fertility

There was no evidence of a statistically significant effect of treatment on mite fertility. There was no statistically significant effect of either treatment group or assay number or the interaction between these two terms in either the conditional or the zero-inflation part of the GLMM of mite fertility.

## Discussion

The purposes of this study were to assess whether an ethogram could be used to measure behaviours indicative of pain/distress in hens undergoing a routine experimental procedure, assess any analgesic effect of EMLA 5% Cream on pain related behaviour using the ethogram and whether the use of EMLA cream affected poultry red mite feeding, mortality and fecundity data collected during a trial.

With regards to mite feeding, the mites fed on hens in the EMLA group showed the lowest mean feeding rates on all days although this was only statistically significantly lower than the no treatment group in the first feeding assay. The effect of the EMLA cream on mites could be physical or chemical and may decrease with time. Its effect on mite feeding on hens that have had a longer period between treatment and feeding assays should be evaluated as well as its effect on the other blood-feeding life stages of the mite. Variation in mite feeding is an issue with ‘farm-collected’ mites with mite fitness dependent on an array of factors not yet understood ( Nunn *et al.*, 2020).

The behaviour categories in the ethogram were based on observations from two scientists with experience of the procedure over the course of several trials including four published trials ( Lima-Barbero *et al.,* 2019a, b; Nunn *et al.,* 2019, 2020). The highest behaviour scores were demonstrated on plucking day, when no hens received treatments and all hens had their left thighs plucked. On treatment day, decreased probabilities of displaying pain-related behaviours were demonstrated in all three experimental groups when compared to plucking day, although this was largest and only statistically significant for the EMLA group, suggesting successful analgesia in this group. Progressive removal of feathers has previously been shown to lead to a decrease in the type of agitated behaviour observed during the first feather removal ( Gentle and Hunter, 1991) and is a reminder that indicators of pain differ between species. A decrease in a certain behaviour does not necessarily mean a decrease in pain/distress if all other factors remain constant and care must be taken in interpretation of results.

Chickens have a range of vocalisations ( Bright, 2008) and the ones scored in this trial were those directly associated with the plucking of feathers and were distinct from the background, low vocalisations witnessed during dummy handling and when the hens were removed from the other hens. Vocalisations were classed on whether they corresponded to having a feather pulled, whether this was repeated more than once or whether it was a distinctive loud squawk. No vocalisations other than the background vocalisations were recorded during the dummy handling session although all birds vocalised on plucking day with 14/15 repeatedly vocalising and 3/15 giving additional single loud vocalisations demonstrating this as a useful measure of pain/distress in birds.

The behaviours that showed the most pronounced increases in estimated probability of occurrence from dummy handling day to the plucking day were blinking and vocalisations. Conversely there was a slight decrease in the estimated probability of moving the leg from the dummy handling day to the plucking and treatment days. The largest estimated differences between treatment groups within a particular day stemmed from lower behaviour counts in the EMLA group on the treatment day.

To allow respiratory rates of the hens to recover from being caught by a handler, sessions were carried out to habituate hens to being caught and taken to a different place away from the rest of the birds. There was no evidence to support a relationship between treatment group and the change in respiration rate before and after the procedure in our data. Nonetheless, the use of respiratory rates in monitoring pain in mammals under anaesthesia is routine practice in some species although there is a lack of published data on respiratory rates on birds and pain. An online respiration guide from The International Veterinary Academy of Pain Management exists, though there are no metadata detailing its development. This scale of 0–3 indicates that a 10% increase in respirations equals a score of zero, with a 10–50% increase leading to a scale of 1, a 2 being 50–100% increase and 3 more than 100% increase. By this scale and using the percentage change in respirations that were demonstrated in this study, all the birds in this study would have scored a zero during dummy handling day, 10 of the birds used would have had a pain score of 1 during plucking day and five birds a pain score of 1 on treatment day. Only birds in the EMLA group (4/5) scored a zero-percentage change in respiratory rate during treatment day. Heart rate monitoring to monitor pain was considered for this trial but is skilled and takes practice ( Smith *et al.*, 2014). Respirations were judged to be a more attainable measurement for those not skilled in auscultation and their assessment as an effective tool in monitoring pain/distress would be useful for other researchers. It is likely that this study did not possess sufficient power to detect an effect of treatment on change in respiration rate if such an effect did exist, particularly as the respiration rate can be affected by outside noise.

Overall significant differences were observed by behavioural scores, indication that use of an ethogram to measure behaviour is useful during experimental procedures. Further work is required to evaluate if learned helplessness occurs across repeated procedures in longitudinal trials and the usefulness of this ethogram to measure behaviour over time. The results presented here indicate measurable differences in behaviour during ‘benign’ procedures such as the dummy handling session in this trial and the regulated procedures in both plucking sessions and that pain relief during the latter is useful in mitigating distress. Previously published studies ( Flecknell *et al.,* 1990; Keating *et al.*, 2012) in the use of EMLA cream as an analgesic in the pain management of laboratory animals exist for small animals (rats, rabbits and dogs) during procedures such as venepuncture and tattooing. It is helpful to think of the alleviation of distress in birds rather than pain given the difficulties in recognising signs of pain across classes, species and in individuals as reviewed by Flecknell (1994). Both the EMLA cream and aqueous cream were easily absorbed, to the point that on removal of bandages, handlers could not tell which birds were in the no treatment group or the EMLA and placebo groups. The creams were easily applied around the feathers using the interdental brushes. The dosage leads to a small volume of product that would not cover a larger surface area (i.e. > 3×4 cm) although it would be enough to use in minor procedures such as blood sampling etc. No adverse effects were demonstrated suggesting that it could be safely used at that dose for other procedures.

Currently there are four active research teams in Europe working in early-stage vaccine development with further groups in Japan that this research may benefit. There are also groups in Europe who have adapted the device for other uses in the study of PRM such as mite genetic studies and behavioural studies that use mite feeding. Research groups using the on-hen mite feeding device will typically use between 4–5 birds per experimental group ( Nunn *et al.,* 2019; Lima-Barbero *et al.,* 2019a, b) leading to a refinement in the procedure for each of those birds.

The study provides a reference point of EMLA use for plucking and potentially other minor procedures in hens used in other experimental settings. Of 1.73 million experimental procedures in 2021, 14% were carried out on birds, although a breakdown of species is not available ( U.K. Government Home Office, 2022) the use of respiratory rates for other species may also prove useful.

Backyard hens are a popular pet species, with an estimated 1.4 million in 2021–2022 ( Bedford, 2022) with ‘chicken friendly’ veterinary practices across the UK that have specialist knowledge of chicken care. The use of EMLA cream in this study provides a reference point for clinicians who may want to provide hens with local analgesia for certain procedures.

In summary, the ethogram used to measure behaviour, demonstrated a significant increase in stress-related behaviours when used during the plucking of feathers from hens’ thighs compared with the benign ‘dummy handling‘session. EMLA cream did significantly reduce the ethogram scores when plucking treated hens, with no adverse effects on hen behaviour during the incubation period.

Application of EMLA cream is an easy, non-invasive method to use and its incorporation in experimental procedures should be encouraged, though effects on other measurements, such as mite feeding on the treated area need to be considered.

## Data Availability

Figshare: Measuring behaviour in hens using an ethogram to assess analgesia during further refinement of a high welfare, on-hen, poultry red mite feeding device. https://doi.org/10.6084/m9.figshare.22183009 ( Nunn, 2023). This project contains the following underlying data:
• Feeding and mortality data_DE.xlsx (raw feeding, mortality and fecundity data collected at timepoints.) • Obj 2 18week Data for Power Calcs.xlsx (feeding data from previous trial use to perfom power calculations for hens of a same age for this trial) • PowerCalculationsNewProject_Feb2021.pdf (power calculation reports) • raw ethogram scores FINAL docx.docx (raw data collected from trial) • Raw Respiration rates.docx.docx (raw data collected from trial) • Supp. File 1 (ethogram).docx (ethogram table listing behaviours) Figshare: ARRIVE checklist for ‘Measuring behaviour in hens using an ethogram to assess analgesia during further refinement of a high welfare, on-hen, poultry red mite feeding device’. https://doi.org/10.6084/m9.figshare.22183009 ( Nunn, 2023). Data are available under the terms of the Creative Commons Attribution 4.0 International license (CC-BY 4.0).

## Footnotes

^1^
On ‘treatment day’ an error reading leg rings meant that two birds had to be reassigned experimental groups. As the birds had already been kept in their groups for over a week, the decision was made to keep them in the same pen hence the uneven distribution of experimental groups in the last pen.
