# Supplementary material for: Measuring behaviour in hens using an ethogram to assess analgesia during further refinement of a high welfare, on-hen, poultry red mite feeding device
Source: F1000Res. 2023 Jun 20;12:715. [Version 1] doi: 10.12688/f1000research.133390.1 (PMC11002522; doi:10.12688/f1000research.133390.1)
Supplement: Peer review history

# Reviewer report: Reviewer response for version 1

**DOI:** 10.5256/f1000research.146376.r205055

**Published:** 2024-04-08

**Authors:** Mike J. McGrew

**Affiliations:** 1 The Roslin Institute and Royal (Dick) School of Veterinary Studies, The University of Edinburgh, Edinburgh, Scotland, UK

This article by Nunn *et al.* addressing the reduction pain after feather plucking is an interesting, well written article that address an important welfare question. The authors design an ethogram to examine behaviour after feather plucking. Breathing rates were monitored and the effect of the analgesic on red mite feeding rates and fertility rates were measured.

 The authors find that using analgesic cream reduced the probability of observing a pain related behaviour after plucking a 3 cm area on the leg.

 My comment is that the plucking/analgesic analysis is focused on red mite and may have broader experimental uses for birds. Feather plucking is also used as a method to obtain feather pulp which is cultured in vitro to obtain fibroblasts from adult birds of many species (Kjelland and Kraemer, 2012 ^1^). The results here indicate that an analgesic cream would be useful in reducing indications of pain after feather plucking. My question is if the cream can be delivered after the procedure to alleviate pain related behaviour would mean the bird needs to be handled twice which might increase bird distress, i.e. the cream needs to be delivered before plucking, and the manufacturer states that the cream be placed under a dressing. Perhaps the authors could further comment on the other uses of feather plucking in the discussion.

 Specific comment:

 I am confused by the Methods section, page 6 (PDF version) and the conditions that occurred on the plucking day, right leg (day 6) and on the treatment day, left leg (day 7).

 From the Methods’ text, it appears no analgesic was used on day 6, analgesic was only used on day 7 for the right leg. Yet in Figure 2, there are three conditions listed for the plucking group (day 6): none, EMLA, placebo as well as three conditions for the treatment group (day 7): none, EMLA, placebo. How is this possible?

Are the 3Rs implications of the work described accurately?

Yes

Is the work clearly and accurately presented and does it cite the current literature?

Yes

If applicable, is the statistical analysis and its interpretation appropriate?

I cannot comment. A qualified statistician is required.

Are all the source data underlying the results available to ensure full reproducibility?

Yes

Is the study design appropriate and is the work technically sound?

Yes

Are the conclusions drawn adequately supported by the results?

Yes

Are a suitable application and appropriate end-users identified?

Yes

Are sufficient details of methods and analysis provided to allow replication by others?

Partly

Reviewer Expertise:

Genetic modification

I confirm that I have read this submission and believe that I have an appropriate level of expertise to confirm that it is of an acceptable scientific standard.

## References

[1] : Feathers and Post-Hatch Eggshells: Sources of Fibroblast Cells for Conserving Genetic Diversity. *Avian Biology Research* .2012;5(3) : 10.3184/175815512X13442766780124 123-130 10.3184/175815512X13442766780124

# Reviewer report: Reviewer response for version 1

**DOI:** 10.5256/f1000research.146376.r196063

**Published:** 2024-01-22

**Authors:** Jan Perner, David Hartmann

**Affiliations:** 1 Institute of Parasitology, Biology Centre, Czech Academy of Sciences, Ceske Budejovice, Czech Republic; 2 Institute of Parasitology, Biology Centre, České Budějovice, Czech Republic

The aim of the study by Nunn *et al.* is to evaluate an ethogram as a proxy for discomfort/distress/pain in hens during procedures, such as feather plucking (for example, before on-animal poultry red mite feeding assays), and to evaluate whether the application of a commercially available short-term local anaesthesia cream EMLA provides any observable relief from pain/distress during routine experimental procedures. Additionally, poultry red mite feeding assays were performed, and the effects of the anaesthesia cream on mite feeding rates, mite mortality, and mite fertility were evaluated.

 In general, the study is interesting and all the conclusions are supported by the presented results.

**Suggestions for improvement of the research article:**

 Abstract, the methods section:

- The description of the three days, when hens’ behaviours were measured, is rather confusing. It is clearly described only later in the text and Table 1. Please consider increasing clarity.
- I recommend replacing 'in the same group' with 'in the EMLA group' to improve understanding.

Introduction:

- I miss a mention of an artificial membrane feeding system for *D.gallinae* mites, which is suitable, at least for screening and initial testing of acaricides, and represents a possibility for a partial replacement of experimental animals.

Results:

- Bodily resistance has zero observations across groups. Possibly consider removing this criterion. The applied statistics also skews slightly the first-hand impression, where not every zero is plotted equally.

Discussion:

- Even though it was not the purpose of this study, I would appreciate if there were more comments on Figure 3 in the discussion. What is the explanation for lower rates in the first feeding of EMLA(Lidocaine and Prilocaine)-treated group? Is it a common observation that feeding rates decline during subsequent feedings (applies for the 'placebo' and 'none' groups). Strikingly, the Aqueous Cream B.P. seems to significantly enhance the third feeding, despite being neglected both in results and discussion. Possibly rectify this overlook if you think it deserves attention. Can this be attributed to enhanced skin physicality?
- Raw ethogram scores are difficult to read as there is no indication of the belonging of particular hens to individual experimental groups. I suggest changing the row order according to experimental groups rather than according to the number on the hens’ label. Furthermore, this table could be moved to supplementary data.

Title:

- The title implicates a combination of ethogram and *D. gallinae* feeding. While the authors define well the former, and then describe the latter, ie the *D. gallinae* feeding as a function of feeding repetition and +/- analgetics or lotion application, the application of ethogram on the variances of *D. gallinae* feeding is a sad miss in this work.

Are the 3Rs implications of the work described accurately?

Yes

Is the work clearly and accurately presented and does it cite the current literature?

Yes

If applicable, is the statistical analysis and its interpretation appropriate?

I cannot comment. A qualified statistician is required.

Are all the source data underlying the results available to ensure full reproducibility?

Yes

Is the study design appropriate and is the work technically sound?

Yes

Are the conclusions drawn adequately supported by the results?

Yes

Are a suitable application and appropriate end-users identified?

Yes

Are sufficient details of methods and analysis provided to allow replication by others?

Yes

Reviewer Expertise:

Molecular Biology of ticks and mites.

We confirm that we have read this submission and believe that we have an appropriate level of expertise to confirm that it is of an acceptable scientific standard.
